# Exploratory Clinicopathological Associations Between Prespecified Clinical–Structural Domains and Synovial Tissue Findings in End-Stage Knee Osteoarthritis

**DOI:** 10.3390/diagnostics16213593

**Published:** 2026-10-30

**Authors:** Lorand Vitalis, Andrei Marian Feier, Sabin Gligore Turdean, Ovidiu Simion Cotoi, Tudor Sorin Pop

**Affiliations:** 1 Doctoral School, George Emil Palade University of Medicine, Pharmacy, Science, and Technology of Targu Mures, 540142 Targu Mures, Romania; dr.vitalis.lorand@gmail.com; 2 Department M4 Clinical Sciences, Orthopedics and Traumatology I, George Emil Palade University of Medicine, Pharmacy, Science, and Technology of Targu Mures, 540139 Targu Mures, Romania; tudor.pop@umfst.ro; 3 Department ME1 Morphological Sciences, Morphopathology, George Emil Palade University of Medicine, Pharmacy, Science, and Technology of Targu Mures, 540139 Targu Mures, Romania; sabin.turdean@umfst.ro; 4 Department M2 Functional and Complementary Sciences, Pathophysiology, George Emil Palade University of Medicine, Pharmacy, Science, and Technology of Targu Mures, 540139 Targu Mures, Romania; ovidiu.cotoi@umfst.ro

**Keywords:** knee OA, clinicopathological associations, synovial histopathology, synovitis, synovial histopathology, TKA, inflammatory domain

## Abstract

**Background**: Knee osteoarthritis (OA) is a heterogeneous whole-joint disease. This exploratory clinicopathological study evaluated associations between four prespecified continuous clinical and structural domains and synovial tissue findings in patients undergoing total knee arthroplasty (TKA) for primary end-stage knee OA. **Methods**: This prospective single-center study included 79 patients undergoing cemented TKA. Standardized inflammatory, metabolic/lipomatous, osteocartilaginous/remodeling, and mechanical domain scores were evaluated against suprapatellar histopathological and intraoperative macroscopic synovial outcomes. Associations were examined using Spearman correlations and age- and sex-adjusted regression models with false-discovery-rate (FDR) correction. **Results**: Low-grade synovitis was present in 63.3% of knees and high-grade synovitis in 31.6%. Unadjusted analyses showed weak correlations between the inflammatory domain and macroscopic synovitis (ρ = 0.24, *p* = 0.032) and between the metabolic/lipomatous domain and synovial lipomatosis severity (ρ = 0.23, *p* = 0.042). Adjusted estimates were β = 0.24 (*p* = 0.043) for inflammatory domain–Krenn synovitis, β = 0.23 (*p* = 0.050) for inflammatory domain–macroscopic synovitis, and OR = 1.57 (*p* = 0.044) for metabolic/lipomatous domain–lipomatosis severity. No associations were observed for the osteocartilaginous/remodeling or mechanical domains. None of the six prespecified associations remained statistically significant after FDR correction. **Conclusions**: End-stage knee OA showed substantial synovial heterogeneity, but no prespecified domain–tissue association remained significant after multiple-testing correction. The evaluated domains should therefore be considered exploratory clinicostructural constructs rather than validated biological or diagnostic classifications. Further multicenter longitudinal studies integrating imaging, molecular biomarkers, and tissue assessment are needed.

## 1. Introduction

Knee OA is a heterogeneous whole-joint disease involving cartilage, subchondral bone, synovium, menisci, ligaments, infrapatellar fat pad, and periarticular tissues [1,2]. Its clinical and biological variability reflects partially overlapping inflammatory, metabolic, structural, and mechanical processes rather than a single uniform disease mechanism [3,4], and patients with similar radiographic severity may therefore differ considerably in their underlying pathobiology. Synovial inflammation is a frequent component of OA and can be identified histologically, macroscopically, and by contrast-enhanced imaging [5,6]. OA synovitis is characterized by lining hyperplasia, inflammatory-cell infiltration, stromal activation, vascular proliferation, and fibrosis [5,7] and has been associated with pain, functional impairment, and structural progression [5,6]. Imaging-pathology studies further demonstrate correlations between MRI-detected synovitis, macroscopic abnormalities, and histological inflammation [8,9], supporting an integrated clinicopathological assessment that may provide information beyond radiographic severity alone. OA stratification frameworks encompass several conceptually distinct constructs. Clinical phenotypes describe observable patient characteristics, whereas endotypes refer to subgroups defined by distinct biological mechanisms [10,11,12]. Structural patterns are primarily based on radiographic or imaging features, while symptom-based domains characterize manifestations such as pain severity, sensitization, or functional impairment. Inflammatory, metabolic, mechanical, and osteocartilaginous processes have been proposed as biologically informed disease dimensions, whereas chronic pain and central sensitization are more appropriately considered symptom- or pain-processing constructs rather than equivalent mechanistic disease endotypes [10,11,12]. Most proposed frameworks have been derived from clinical characteristics, imaging, biochemical biomarkers, pain profiles, or combinations of these variables [10,11]. Current recommendations emphasize that observable clinical phenotypes, structural patterns, molecular endotypes, symptom-based domains, prognostic groups, and treatment-response subgroups represent distinct levels of stratification and should not be used interchangeably [12]. In the present study, a domain refers to a prespecified continuous composite score summarizing related clinical or structural variables, whereas a profile refers only to the secondary categorical assignment derived from these domain scores. The terms phenotype and endotype are reserved for the literature, with phenotype referring to an observable patient subtype and endotype to a subgroup defined by a distinct biological mechanism [12,13]. Histopathological assessment of synovium obtained during TKA provides direct tissue-level information complementary to clinical, radiographic, and biomarker data [7,14]. Although the Krenn score standardizes lining hyperplasia, stromal cellularity, and inflammatory infiltrates, OA synovium also demonstrates vascular, fibrotic, lipomatous, calcific, and metaplastic changes, reflecting substantial morphological heterogeneity [7,14,15]. Integrating histological, macroscopic, radiographic, laboratory, and clinical findings within the same surgical cohort may therefore provide a broader clinicopathological assessment than any single data source alone [8,9,12]. The primary aim of this exploratory study was to evaluate whether four prespecified continuous clinical and structural domain scores were associated with independent histopathological and macroscopic synovial tissue findings in patients undergoing cemented TKA for primary end-stage knee OA.

## 2. Materials and Methods

### 2.1. Study Design

This was a single-center prospective observational clinicopathological study including patients who underwent cemented TKA for primary end-stage knee OA at the Department of Orthopedics and Traumatology of the Clinical County Hospital of Mureș, Romania, between 25 July 2025 and 31 March 2026. The study evaluated whether four prespecified clinical and structural domains—(a) inflammatory, (b) metabolic/lipomatous, (c) osteocartilaginous/remodeling, and (d) mechanical—were associated with histopathological and macroscopic synovial findings. The primary analytical objective was to examine continuous domain–tissue outcome associations. Domain scores were derived exclusively from clinical, laboratory, radiographic, and non-synovial structural variables, whereas microscopic and macroscopic synovial findings were treated as tissue-level outcomes. The study was reported in accordance with the STROBE statement. The study protocol was approved by the Ethics Committee of the Clinical County Hospital of Mureș on 22 July 2025 (approval code: 10918) and was conducted in accordance with the Declaration of Helsinki. Written informed consent for participation and tissue collection was obtained from all participants.

### 2.2. Study Setting and Data Sources

The study was conducted in the Department of Orthopedics and Traumatology in collaboration with the Department of Pathology. Eligible patients were identified prospectively from the operative schedule and screened before surgery. Demographic and clinical data were obtained from preoperative assessments and hospital records, while laboratory values were obtained from blood tests performed no more than 21 days before surgery. Radiographic and structural data were obtained from preoperative imaging and operative documentation. Dedicated preoperative assessment of synovitis by ultrasound or MRI was not included in the study protocol, as these examinations are not routinely obtained for surgical planning in patients with established end-stage radiographic knee OA. Histopathological findings were derived from protocol-based synovial tissue assessment. Macroscopic synovial features were recorded intraoperatively using a standardized assessment form, with intraoperative photographs. All data were linked at the operated-knee level and integrated to examine associations between routinely accessible clinical and structural information and independent synovial tissue findings.

### 2.3. Participants and Eligibility Criteria

All consecutive adults scheduled for primary TKA at the Department of Orthopedics and Traumatology between 25 July 2025 and 31 March 2026 were screened prospectively for eligibility. Cemented fixation represented the routine primary TKA technique at our institution during the study period; uncemented TKA was not routinely performed in the public hospital setting. Screening was performed during the first preoperative assessment by a member of the study team using the operative schedule and hospital records. Patients were included in the analytical cohort when the following criteria were met: age ≥ 40 years; primary end-stage knee OA with Ahlbäck grade IV or V; recommendation for primary TKA by a senior orthopedic surgeon; completion of primary cemented TKA at our institution; adequate synovial tissue for histopathological assessment; available preoperative clinical data; and laboratory results obtained within 21 days before surgery. Patients with post-traumatic OA, inflammatory arthritis, systemic connective tissue disease, active or previous septic arthritis, active knee infection, malignant joint disease, previous extensive synovectomy, intra-articular injection within the preceding 12 months, or known crystal arthropathy involving the operated knee were excluded. The operated knee represented the unit of analysis; in bilateral cases, only the first operated knee was included.

### 2.4. Clinical, Laboratory, Radiographic, and Surgical Variables

Demographic and clinical variables included age, sex, body mass index, symptom duration, VAS pain score, relevant comorbidities, previous knee surgery, and medication use. Variables included in the (a) inflammatory domain were C-reactive protein, erythrocyte sedimentation rate, fibrinogen, and neutrophil-to-lymphocyte ratio. These variables were selected as routinely available systemic inflammatory indicators and were not intended to constitute specific molecular biomarkers of a synovial inflammatory endotype. Variables included in the (b) metabolic/lipomatous domain were body mass index, glycated hemoglobin, triglycerides, low-density lipoprotein cholesterol, and high-density lipoprotein cholesterol. BMI was the only direct anthropometric measure available, while HbA1c and the lipid variables were included to capture complementary aspects of systemic metabolic burden. Variables included in the (c) osteocartilaginous/remodeling domain were radiographic disease severity according to the Ahlbäck classification, osteophyte burden, subchondral sclerosis, extent of cartilage loss, and number of compartments involved. The Ahlbäck classification is the established radiographic grading system for advanced knee OA routinely applied in our orthopedic department. Variables included in the (d) mechanical domain were absolute hip–knee–ankle deviation, compartment-specific overload, meniscal deficiency, and focal cartilage defect. Alignment direction (varus or valgus) and predominant compartment involvement were recorded for descriptive characterization but were not included in the domain-score calculations. Age, sex, pain, and symptom duration were used for cohort characterization but were not included in the domain scores; age and sex were used for covariate adjustment. A detailed description of definitions, codes, and data sources is presented in Supplementary Table S1.

### 2.5. Histopathological and Macroscopic Synovial Assessment

Synovial tissue was collected intraoperatively from the suprapatellar pouch. Additional specimens were obtained from macroscopically abnormal synovium when present. Patient-level Krenn scores, individual histopathological-feature grades, and composite histopathological scores were derived from the suprapatellar specimen. Tissue samples were fixed in 10% neutral-buffered formalin, paraffin-embedded, sectioned at 4 µm thickness, and stained with hematoxylin and eosin. Samples without adequate synovial lining or subsynovial tissue were excluded from histopathological evaluation. Histopathological assessment was performed using a scoring sheet by a single pathologist who was blinded to the clinical, laboratory, and radiographic data. Synovitis severity was evaluated using the Krenn synovitis score [15], comprising synovial lining hyperplasia, stromal cellularity, and inflammatory infiltrate, each graded from 0 to 3. Total scores of 0–1, 2–4, and 5–9 were classified as absent, low-grade, and high-grade synovitis, respectively. Additional histopathological features, including villous hyperplasia, vascular proliferation or congestion, fibrosis, lipomatosis, calcification, chondrometaplasia, chondromatosis-like change, fibrin or fibrinoid deposition, hemosiderin-rich macrophages, and reactive synovial changes, were assessed using predefined semiquantitative ordinal scales. Features were graded semiquantitatively as absent (0), mild (1), moderate (2), or marked (3). The synovial remodeling score was calculated as the sum of the ordinal scores for fibrosis, calcification, and chondrometaplasia, resulting in a score ranging from 0 to 9, with higher values indicating greater structural synovial remodeling. Macroscopic synovial assessment was performed intraoperatively by the operating orthopedic surgeon using a standardized evaluation form. Synovial hypertrophy, vascularity, villous appearance, pigmentation, and nodularity were graded as absent (0), mild (1), moderate (2), or marked (3). The macroscopic synovitis score was calculated as the sum of the hypertrophy, vascularity, and villous-appearance scores, resulting in a theoretical range of 0–9. Pigmentation and nodularity were reported separately. Global macroscopic synovitis was recorded separately as absent, mild, moderate, or marked based on the intraoperative appearance. The additional semiquantitative histopathological and macroscopic grading scales were study-defined and were not previously validated as formal scoring systems. They were applied using predefined ordinal categories (absent, mild, moderate, or marked) to standardize morphological assessment across specimens. Intraobserver reproducibility was assessed by repeat blinded evaluation of a subset of 30 specimens after a washout interval of 6 months. Agreement for the semiquantitative histopathological and macroscopic assessments was evaluated using weighted Cohen’s kappa and intraclass correlation coefficients, as appropriate; detailed results are presented in Supplementary Table S3.

### 2.6. Clinical and Structural Domain Scores

The conceptual composition of the four domains, the variables assigned to each domain, and the direction of scoring were defined before examination of associations with the synovial tissue outcomes. No component variable was selected, removed, or weighted according to its observed association with histopathological or macroscopic synovial findings. Cohort data were used only to standardize variables to a common scale and to calculate the resulting domain scores. Continuous variables assigned to each domain were standardized as z-scores by subtracting the cohort mean and dividing by the standard deviation. Ordinal variables were entered according to their predefined numerical grades, and binary variables were coded as 0 or 1 before standardization. Each component contributed equally to its corresponding domain. Equal weighting was used as a transparent aggregation rule in the absence of an externally validated differential weighting scheme. Outcome-derived weights were deliberately not estimated from the present cohort to avoid overfitting. The inflammatory domain score was calculated as the arithmetic mean of the standardized values of C-reactive protein, erythrocyte sedimentation rate, fibrinogen, and neutrophil-to-lymphocyte ratio. Accordingly, the inflammatory domain was interpreted as a composite measure of routine systemic inflammatory burden. The metabolic/lipomatous domain score was calculated from body mass index, glycated hemoglobin, triglycerides, low-density lipoprotein cholesterol, and high-density lipoprotein cholesterol. High-density lipoprotein cholesterol was reverse-coded before averaging so that higher scores represented greater metabolic burden. This domain should be interpreted as a composite of routinely available anthropometric and metabolic variables and not a specific measure of central or visceral adiposity. The osteocartilaginous/remodeling domain score was calculated from Ahlbäck grade, osteophyte burden, subchondral sclerosis, extent of cartilage loss, and number of compartments involved. The mechanical domain score was calculated from absolute hip–knee–ankle deviation, compartment-specific overload, meniscal deficiency, and focal cartilage defect. After calculation, each composite domain score was re-standardized across the cohort to a mean of 0 and a standard deviation of 1. These re-standardized scores were used for cross-domain comparison and descriptive profile classification. Domain scores were calculated only for participants with complete data for all components of the respective domain. Continuous domain scores represented the primary analytical exposures. Descriptive profile classification was performed secondarily as a methodological illustration of the distribution and overlap of the four domain scores. The categorical profile classification was derived secondarily from these continuous domain scores using study-defined thresholds and was intended only to illustrate patterns of domain predominance and overlap. Because these profiles were generated from the same cohort-standardized scores, they should not be interpreted as independently derived or biologically validated phenotypes or endotypes. A predefined hierarchical algorithm was used for this descriptive classification. A domain was considered elevated when its re-standardized score was at least 0.50 standard deviations above the cohort mean. A participant was classified as having a mixed profile when the two highest domain scores were both at least 0.50 and differed by less than 0.40 standard deviations. Participants whose mechanical score was highest were also classified as mixed when either the inflammatory or metabolic/lipomatous score was at least 0.50, irrespective of the difference between the scores. Among participants not meeting the mixed-profile criteria, the highest elevated score determined the dominant inflammatory, metabolic/lipomatous, osteocartilaginous/remodeling, or mechanical profile. A dominant mechanical profile was termed a low-inflammatory mechanical profile only when both the inflammatory and metabolic/lipomatous scores were below 0.50. Participants without any domain score of at least 0.50 were marked as unclassified.

### 2.7. Outcomes

The primary endpoints were the associations between the continuous clinical and structural domain scores and their corresponding independent synovial tissue outcomes: (1) inflammatory domain–Krenn synovitis score; (2) inflammatory domain–macroscopic synovitis score; (3) metabolic/lipomatous domain–synovial lipomatosis severity; (4) osteocartilaginous/remodeling domain–synovial remodeling score; (5) mechanical domain–Krenn synovitis score; and (6) mechanical domain–macroscopic synovitis score. These tissue outcomes were selected as independent measures for the exploratory evaluation of clinicopathological associations and were not treated as reference standards for formal diagnostic accuracy analysis.

Secondary tissue endpoints included the individual Krenn components, villous hyperplasia, vascular proliferation, fibrin or fibrinoid change, fibrosis, calcification, chondrometaplasia, chondromatosis-like change, siderophages, synovial hypertrophy, macroscopic vascularity, and global macroscopic synovitis. Descriptive comparisons among the categorical profile groups were considered secondary exploratory analyses. Postoperative clinical follow-up included VAS pain and Lower Extremity Functional Scale (LEFS) assessments at 1, 3, and 6 months after TKA. These outcomes were considered exploratory measures of short-term postoperative recovery and were not part of the primary clinicopathological endpoint framework. The 6-month visit represented the longest available follow-up assessment.

### 2.8. Statistical Analysis

Continuous variables were summarized as mean ± standard deviation or median [interquartile range], as appropriate, and categorical variables as frequencies and percentages. Domain scores were analyzed as continuous standardized exposures. Unadjusted associations with synovial outcomes were assessed using Spearman correlation. Age- and sex-adjusted associations were evaluated using linear regression for the Krenn synovitis, macroscopic synovitis, and synovial remodeling scores, and ordinal logistic regression for synovial lipomatosis severity. Age, sex, relevant comorbidities, smoking status, and concomitant medication use were also compared across the descriptive profile groups to assess potential demographic and clinical imbalance. Standardized regression coefficients or odds ratios with 95% confidence intervals were reported, as appropriate. Model assumptions were assessed before interpretation. The Benjamini–Hochberg procedure was applied separately to the six unadjusted and six adjusted prespecified primary domain–tissue endpoint tests. Before FDR correction, *p*-values < 0.05 were considered nominal, whereas false-discovery-rate-adjusted q-values < 0.05 were considered statistically significant. Descriptive comparisons of tissue outcomes among the four dominant profile groups were performed using one-way analysis of variance or the Kruskal–Wallis test for continuous variables and the chi-square test or Fisher’s exact test for categorical variables. Participants with mixed or unclassified profiles were excluded from these tissue-outcome comparisons but retained in analyses using continuous domain scores. Comparisons of demographic characteristics, comorbidities, smoking status, and medication use included all descriptive profile groups, including the mixed group. As a sensitivity analysis for potential confounding, the six primary continuous domain–tissue associations were re-estimated using parsimonious multivariable models incorporating additional clinically relevant covariates selected according to each domain, while avoiding adjustment for variables directly incorporated into the corresponding domain score. Benjamini–Hochberg FDR correction was applied across the six sensitivity-model tests. Complete-case analyses were performed. No formal a priori sample-size calculation was undertaken because this was an exploratory clinicopathological association study. A post hoc detectable-effect analysis was performed to characterize the magnitude of association that could reasonably be detected with the available sample. With 79 participants, a two-sided α level of 0.05, and 80% statistical power, the study could detect a correlation of approximately ρ = 0.31 or greater for the prespecified domain-outcome associations. Smaller associations may therefore not have been reliably detectable in this cohort. This sensitivity estimate was based on the nominal significance level because the rejection threshold under the Benjamini–Hochberg false-discovery-rate procedure is data-dependent. Changes in postoperative VAS and LEFS scores across the preoperative, 1-month, 3-month, and 6-month assessments were evaluated using the Friedman test for repeated measurements. Exploratory associations of baseline domain scores and synovial tissue measures with 6-month VAS and LEFS were assessed using linear regression models adjusted for the corresponding preoperative outcome, age, and sex. Benjamini–Hochberg FDR correction was applied across the exploratory postoperative association tests. These longitudinal analyses were considered exploratory. Analyses were conducted using IBM SPSS Statistics for Windows, version 23.0.

## 3. Results

**Figure 1:**
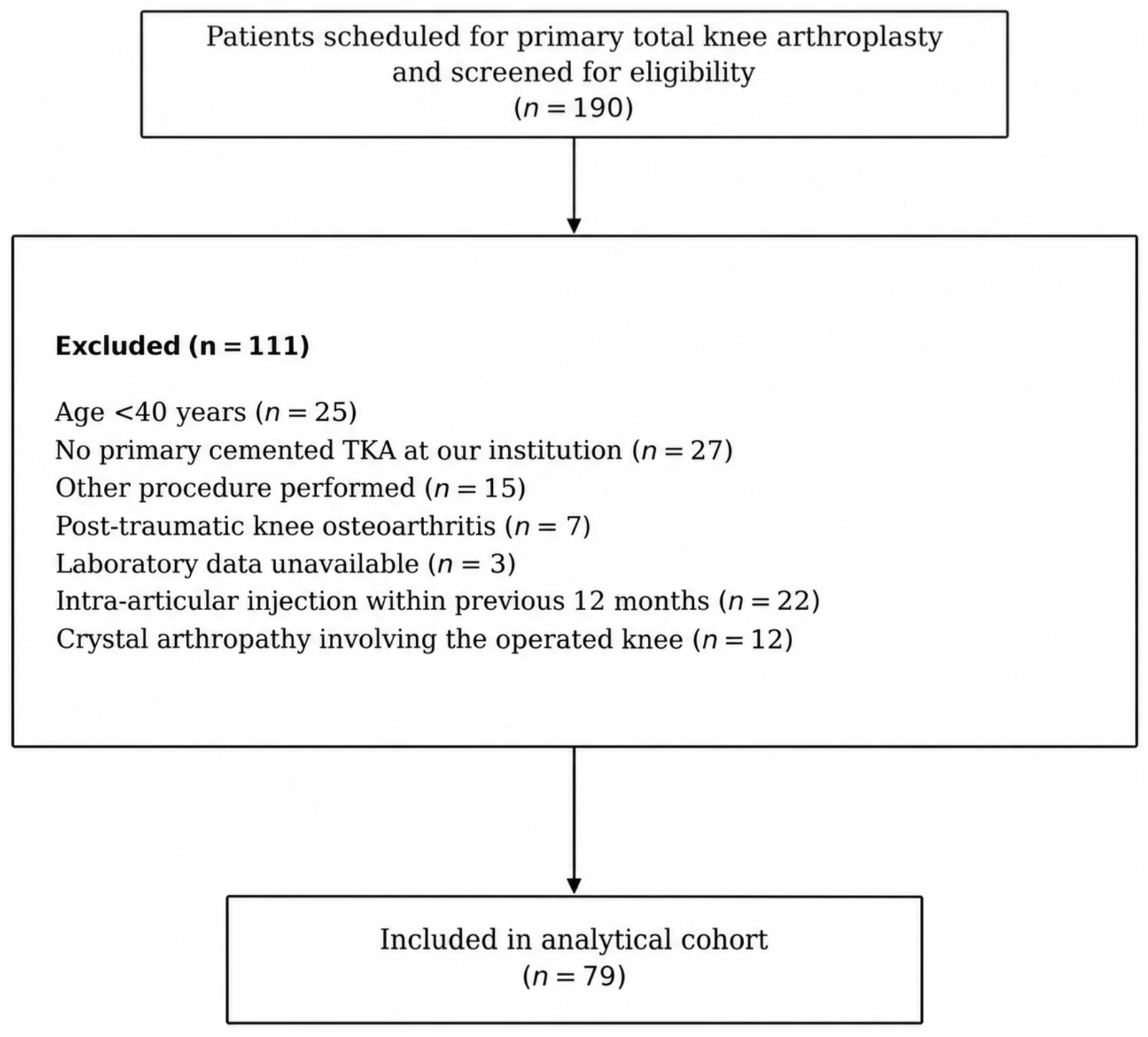
Study flow diagram showing participant screening, exclusions, and final cohort.

A total of 190 patients were screened for eligibility during the study period. Of these, 111 were excluded, and 79 patients were included in the final cohort (Figure 1).

The mean age was 61.5 ± 8.8 years (range, 40–83 years). Four patients (5.1%) were younger than 50 years, 18 (22.8%) were younger than 55 years, and 33 (41.8%) were younger than 60 years; conversely, 46 patients (58.2%) were aged 60 years or older. Women comprised 57.0% of the cohort. The mean body mass index was 30.0 ± 2.9 kg/m^2^. The median symptom duration was 33 (23.5–39.0) months, and the median preoperative VAS pain score was 7 (6–7). Hypertension was present in 44 patients (55.7%), diabetes mellitus in 10 (12.7%), and dyslipidemia in 22 (27.8%). Radiographically, twenty-eight knees (35.4%) were classified as Ahlbäck grade IV and 51 (64.6%) as grade V. The mean absolute hip–knee–ankle deviation was 6.2 ± 2.4 degrees. Varus alignment was present in 51 knees (64.6%) and valgus alignment in 28 (35.4%). The medial compartment was predominantly involved in 48 knees (60.8%), the lateral compartment in 25 (31.6%), and the patellofemoral compartment in six (7.6%). Baseline radiographic and laboratory characteristics are summarized in Table 1 and Table 2.

Laboratory assessment showed a median C-reactive protein level of 4.9 [2.6–7.5] mg/L and a median neutrophil-to-lymphocyte ratio of 2.07 [1.65–2.46]. The mean erythrocyte sedimentation rate was 16.7 ± 7.7 mm/h, and the mean fibrinogen concentration was 3.24 ± 0.47 g/L. The metabolic variables included a median HbA1c level of 5.4 [5.2–5.7]%, mean LDL and HDL cholesterol levels of 105.7 ± 20.2 mg/dL and 52.2 ± 6.6 mg/dL, respectively, and a median triglyceride level of 136 [104–167] mg/dL. The inflammatory and metabolic biomarkers used to calculate the domain scores are summarized in Table 2, with data sources provided in Supplementary Table S1.

Histopathological assessment was available for all 79 knees. The median Krenn synovitis score was 4 [3–5]. Synovitis was absent in 4 knees (5.1%), low-grade in 50 (63.3%), and high-grade in 25 (31.6%). The median synovial remodeling score was 4 [3–5]. Moderate-to-marked fibrosis was present in 41 specimens (51.9%), while moderate-to-marked villous hyperplasia and vascular proliferation were observed in 37 (46.8%) and 36 (45.6%), respectively. Moderate-to-marked lipomatosis was identified in 29 specimens (36.7%), calcification in 26 (32.9%), and chondrometaplasia in 21 (26.6%). Chondromatosis-like changes were present in 10 specimens (12.7%), whereas fibrin or fibrinoid changes and hemosiderin-rich findings were less frequent, occurring in 15 (19.0%) and 3 (3.8%), respectively. Macroscopic synovial assessment was available for all 79 knees, and intraoperative photographs were available for 77 (97.5%). The median macroscopic synovitis score was 4 [3–5]. Moderate-to-marked vascularity was observed in 42 knees (53.2%), villous appearance in 37 (46.8%), global macroscopic synovitis in 35 (44.3%), and synovial hypertrophy in 30 (38.0%). Moderate-to-marked pigmentation was identified in only 2 knees (2.5%), while no moderate-to-marked nodular changes were recorded. Based on the predefined descriptive classification algorithm, 22 patients (27.8%) were classified as having a dominant inflammatory profile, 17 (21.5%) as having a dominant metabolic/lipomatous profile, 19 (24.1%) as having a dominant osteocartilaginous/remodeling profile, and 15 (19.0%) as having a low-inflammatory mechanical profile. Six patients (7.6%) met the criteria for a mixed profile. No participant remained unclassified. Demographic characteristics, relevant comorbidities, and concomitant medication use across the descriptive profile groups are presented in Supplementary Table S4. No statistically significant between-group differences were observed for age, sex, hypertension, diabetes mellitus, dyslipidemia, current smoking, NSAID use, statin use, antidiabetic medication, antiplatelet therapy, or anticoagulant use (all *p* > 0.05). Histopathological and macroscopic synovial findings were not used in domain-score calculation or profile classification. As the descriptive profile groups were generated from the same variables used to construct the four domain scores, the standardized component distributions of the four dominant groups are presented in Figure S1. These should not be interpreted as independent validation of biologically distinct phenotypes.

The inflammatory profile group showed the highest mean standardized values for C-reactive protein, erythrocyte sedimentation rate, fibrinogen, and neutrophil-to-lymphocyte ratio. The metabolic/lipomatous profile group showed higher body mass index, glycated hemoglobin, triglyceride, and low-density lipoprotein cholesterol values and lower high-density lipoprotein cholesterol values. The osteocartilaginous/remodeling profile group showed higher standardized values for Ahlbäck grade, osteophyte burden, subchondral sclerosis, cartilage loss, and number of compartments involved. The low-inflammatory mechanical profile group showed higher values for absolute hip–knee–ankle deviation, compartment-specific overload, meniscal deficiency, and focal cartilage defects.

In unadjusted analyses, the inflammatory domain score was positively correlated with macroscopic synovitis (ρ = 0.24, *p* = 0.032), while the metabolic/lipomatous domain score was positively correlated with synovial lipomatosis severity (ρ = 0.23, *p* = 0.042). The association between the inflammatory domain score and the Krenn synovitis score was not statistically significant (ρ = 0.17, *p* = 0.128). No significant associations were observed between the osteocartilaginous/remodeling domain score and the synovial remodeling score (ρ = 0.01, *p* = 0.916), or between the mechanical domain score and either Krenn synovitis (ρ = −0.09, *p* = 0.453) or macroscopic synovitis (ρ = 0.11, *p* = 0.314). None of the six associations remained statistically significant after false-discovery-rate correction.

In age- and sex-adjusted linear regression models, the inflammatory domain score was associated with a higher Krenn synovitis score (standardized β = 0.24, 95% CI 0.01 to 0.46; *p* = 0.043), while the estimate for macroscopic synovitis was standardized β = 0.23 (95% CI 0.00 to 0.45; *p* = 0.050). In ordinal logistic regression, each one-standard-deviation increase in the metabolic/lipomatous domain score was associated with higher odds of greater synovial lipomatosis severity (OR = 1.57, 95% CI 1.01 to 2.42; *p* = 0.044). The osteocartilaginous/remodeling domain score was not associated with the synovial remodeling score (standardized β = −0.06, 95% CI −0.29 to 0.17; *p* = 0.605). The mechanical domain score was not associated with either Krenn synovitis (standardized β = −0.10, 95% CI −0.34 to 0.14; *p* = 0.412) or macroscopic synovitis (standardized β = 0.18, 95% CI −0.05 to 0.42; *p* = 0.122). None of the six adjusted associations remained statistically significant after false-discovery-rate correction. Parsimonious sensitivity analyses produced broadly similar estimates for most associations, with none remaining significant after FDR correction (Supplementary Table S5). Complete unadjusted and age- and sex-adjusted associations between the prespecified domain scores and synovial tissue outcomes are presented in Table 3.

**Figure 2:**
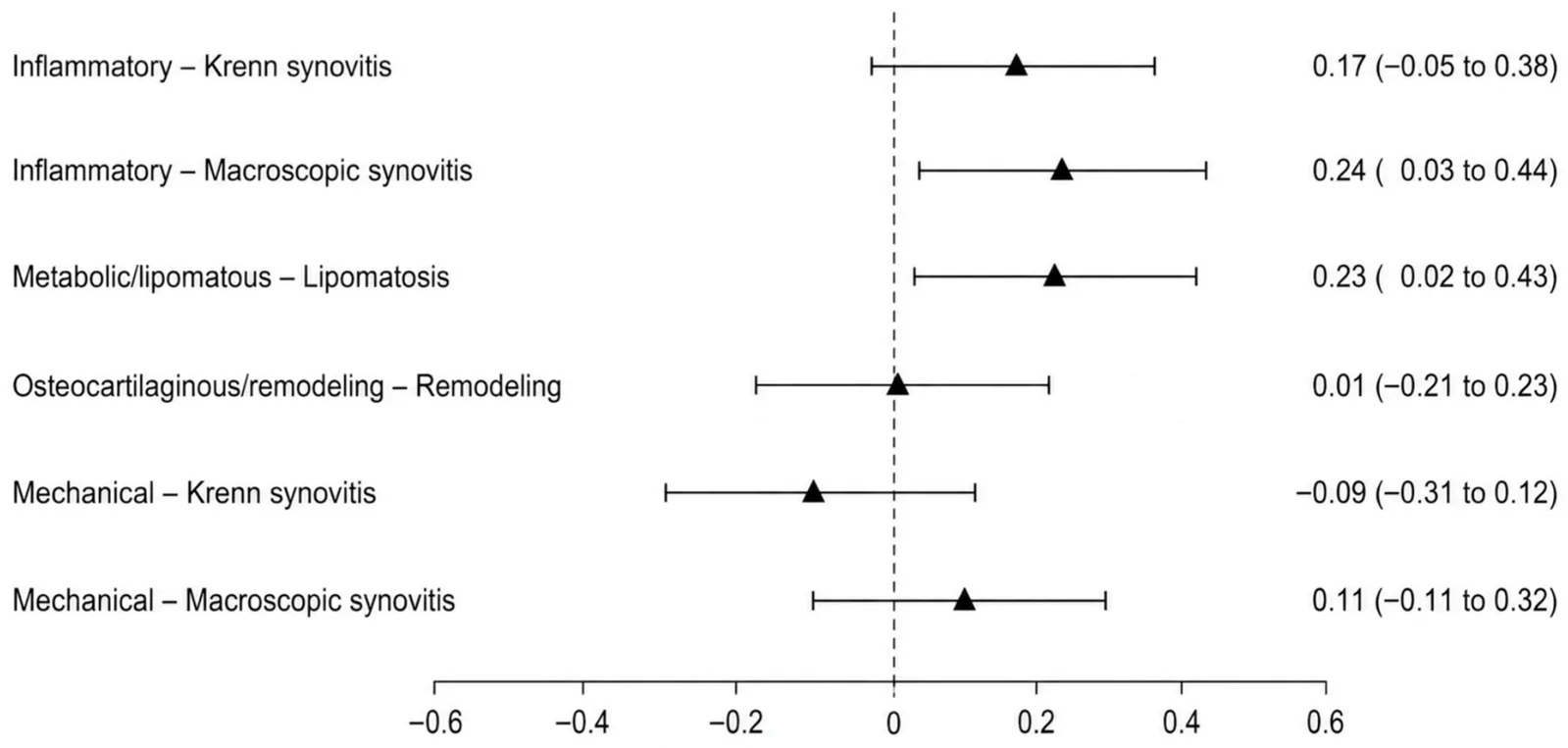
Unadjusted associations between prespecified clinical and structural domain scores and synovial tissue outcomes. Points represent Spearman correlation coefficients, and horizontal lines represent 95% confidence intervals. The dashed vertical line indicates no correlation (ρ = 0).

Descriptive comparisons among the four dominant profile groups, excluding the six participants with mixed profiles, indicated considerable overlap in tissue findings. The inflammatory group had a median Krenn score of 5, compared with 3 in the low-inflammatory mechanical group. The corresponding median macroscopic synovitis scores were 5 and 4, respectively. The metabolic/lipomatous group had a median lipomatosis score of 1.5, while the osteocartilaginous/remodeling group had a median synovial remodeling score of 5. However, the overall between-group differences were not statistically significant for Krenn synovitis (*p* = 0.200), macroscopic synovitis (*p* = 0.137), synovial lipomatosis (*p* = 0.482), or synovial remodeling (*p* = 0.176). The direction and magnitude of the associations between the clinical and structural domain scores and their corresponding synovial tissue outcomes are shown in Figure 2.

Short-term postoperative follow-up demonstrated progressive improvement in both pain and lower-extremity function. Mean VAS scores changed from 6.80 ± 1.02 preoperatively to 4.26 ± 1.18, 2.48 ± 1.25, and 1.74 ± 0.96 at 1, 3, and 6 months, respectively (overall *p* < 0.001). Mean LEFS scores changed from 30.56 ± 8.16 preoperatively to 37.75 ± 8.07, 52.01 ± 8.78, and 61.42 ± 7.54 at 1, 3, and 6 months, respectively (overall *p* < 0.001). Complete longitudinal results are presented in Supplementary Table S2A.

In exploratory postoperative analyses, baseline-adjusted models controlling for age and sex showed a nominal association between the mechanical domain and lower VAS pain at 6 months (standardized β = −0.23, 95% CI −0.46 to −0.01; *p* = 0.039), but this association did not remain significant after false-discovery-rate correction (q = 0.626). No significant adjusted associations were observed for LEFS at 6 months (Supplementary Table S2B).

Representative synovial histopathological sections are provided in Supplementary Figure S2 for illustrative purposes.

## 4. Discussion

This prospective clinicopathological study evaluated associations between four prespecified clinical and structural domains and independent synovial tissue findings in patients undergoing TKA for primary end-stage knee OA. Despite uniformly advanced structural disease, substantial microscopic and macroscopic synovial heterogeneity was observed. The inflammatory and metabolic/lipomatous domains showed nominal exploratory associations with their prespecified tissue outcomes, but none remained significant after false-discovery-rate correction, while the osteocartilaginous/remodeling and mechanical domains showed no significant associations. The marked overlap between descriptive profile groups further indicated insufficient tissue-level discrimination to support validated biological phenotypes or diagnostic categories, consistent with recommendations that OA phenotypes and endotypes require independent validation before clinical application [12,13]. Categorical profiles should be regarded as descriptive summaries of overlapping domain patterns rather than as biologically distinct or validated OA phenotypes.

The descriptive profile classification should be interpreted cautiously because the groups were generated from the same clinical, laboratory, radiographic, and structural variables used to construct the domain scores. Accordingly, the separation shown in Figure S1 is partly algorithmically expected and does not independently demonstrate distinct biological phenotypes. The independent synovial tissue outcomes provide the more relevant clinicopathological comparison, and their overlap and nonsignificant between-group differences indicate limited tissue-level separation.

Synovial findings illustrated substantial tissue heterogeneity despite uniformly advanced OA, with low-grade synovitis in 63.3% and high-grade synovitis in 31.6% of cases. Although the Krenn score captures lining hyperplasia, stromal cellularity, and inflammatory infiltration, OA synovium also exhibits vascular, fibrotic, adipose, and metaplastic changes [14,16]. In the present cohort, these features occurred in varying combinations, consistent with previous arthroplasty and biopsy studies showing variable inflammatory burden and coexistence of inflammatory and remodeling changes in advanced OA [6,13,16,17,18,19,20,21]. This overlap likely reflects simultaneous responses to structural damage, altered loading, metabolic influences, angiogenesis, and repair [16] and further supports interpreting synovial pathology as a continuum rather than as discrete tissue phenotypes.

Regional sampling partly explains the limited correspondence between clinical domains and tissue outcomes. Although standardized suprapatellar sampling reduced variability, synovitis remains spatially heterogeneous within individual knees [22]. This spatial mismatch may have contributed particularly to the nonsignificant mechanical and osteocartilaginous/remodeling associations, because compartment-specific loading, meniscal pathology, cartilage defects, and osteochondral abnormalities may not be reflected by synovial changes sampled from a single suprapatellar site. Macroscopic synovial assessment, histopathology, and contrast-enhanced MRI interrogate related but distinct dimensions of synovial pathology and should not be considered interchangeable measures [23,24,25]. In the present study, the inflammatory domain showed the most consistent nominal association pattern, involving macroscopic synovitis and, after adjustment, Krenn synovitis, although none of these associations remained significant after correction for multiple comparisons. This limited concordance is compatible with the modest specificity of circulating inflammatory biomarkers in OA. Serum hsCRP has been associated with local synovial inflammatory activity [26] but has limited discriminatory performance as a stand-alone marker of local inflammatory activity in OA [27]. By contrast, joint-level biomarkers, including synovial-fluid IL-6 and macrophage-related biomarker panels, may more closely reflect local inflammatory processes [28,29,30]. Imaging-defined synovitis has also been associated with pain and structural progression [31,32,33,34]. Collectively, these findings suggest that routine systemic inflammatory variables provide only an incomplete representation of synovial inflammatory activity in end-stage knee OA.

The metabolic/lipomatous domain showed nominal associations with synovial lipomatosis, but these did not remain significant after multiple-testing correction or produce clear tissue-level separation among descriptive profiles. A metabolic contribution to OA remains biologically plausible, although associations with metabolic syndrome are partly influenced by BMI and other obesity-related factors [35,36,37,38]. The infrapatellar fat pad and adjacent synovium form an immunometabolically active unit, with obesity-related inflammatory and fibrotic changes described in both tissues [39,40,41,42]. However, direct evidence linking routine metabolic variables to histological synovial lipomatosis is limited, and synovial fat deposition in advanced OA may reflect secondary remodeling rather than a specific metabolic marker [43,44,45]. The metabolic/lipomatous domain did not include waist circumference or other direct measures of central or visceral adiposity and should be interpreted as a composite of routinely available metabolic variables and not as a validated metabolic phenotype. The absence of significant osteocartilaginous/remodeling and mechanical associations should not be interpreted as evidence of biological independence, as synovial inflammation has been linked to structural abnormalities, mechanical loading, meniscal pathology, and local osteochondral activity [19,46,47,48,49,50]. In the present study, these relationships may have been attenuated by the spatial mismatch between compartment-specific abnormalities and a single suprapatellar tissue sample, together with the restricted structural variability resulting from inclusion of only Ahlbäck grades IV-V [51,52,53]. Accordingly, the continuous domain scores should be regarded as overlapping clinicostructural constructs, while the categorical profiles remain secondary descriptive classifications rather than validated biological phenotypes [12,54,55,56,57,58].

Strengths of the study include prospective data collection, standardized suprapatellar sampling, and integration of clinical, laboratory, radiographic, operative, macroscopic, and histological data from the same knee. Synovial outcomes were excluded from domain-score construction, continuous scores were retained as the primary exposures, and categorical profiles were treated as secondary descriptive analyses. Reporting of adjusted estimates, effect sizes, confidence intervals, and FDR-corrected results reduced the risk of overinterpreting nominal exploratory associations [59].

Several limitations remain. This was a modest, single-center cohort restricted to end-stage OA, limiting generalizability and structural variability. Although the mean age was relatively young for an end-stage TKA cohort, most patients (58.2%) were aged ≥60 years, and only 5.1% were younger than 50 years. The relatively large proportion of patients younger than 60 years should nevertheless be considered when generalizing these findings to older arthroplasty populations. The cross-sectional design cannot establish causality, and complete-case analysis can introduce selection effects. In addition, although the domain components were prespecified, standardization of component variables and the thresholds used for descriptive profile classification were derived from the present cohort. The resulting domain scores and profile assignments are therefore cohort-dependent and have not been externally validated, which limits their direct generalizability to other populations. The profiles were derived from the same variables as the domain scores and do not validate biological subgroups. Although age and sex were included as covariates in the primary domain–tissue models and no significant demographic, comorbidity, or medication imbalances were identified across the descriptive profile groups, residual confounding by comorbidities and concomitant treatments remains possible. Given the modest cohort size and small profile subgroups, extensive profile-specific multivariable adjustment was not undertaken to avoid overfitting. With 79 participants, the study had approximately 80% power to detect correlations of about |ρ| = 0.31 or greater at a nominal two-sided α of 0.05, so smaller associations may have remained undetected; none of the six prespecified associations remained significant after FDR correction. Circulating cytokines/chemokines and corresponding synovial tissue expression were not measured; the inflammatory domain should be interpreted solely as an exploratory composite of routine systemic inflammatory markers. Although VAS and LEFS improved through 6 months, these data characterize short-term postoperative recovery and do not establish the prognostic value of the clinical and structural domains. The absence of standardized preoperative ultrasound or MRI assessment of synovitis is an additional limitation. Future studies should integrate anatomically matched imaging, synovial tissue assessment, and more specific synovial-fluid or molecular biomarkers. Longitudinal follow-up is needed to determine whether reproducible associations emerge with progression, symptoms, postoperative outcomes, or treatment response [12,56,60].

## 5. Conclusions

In this end-stage knee OA cohort, synovial findings were heterogeneous, but none of the six prespecified domain–tissue associations remained statistically significant after false-discovery-rate correction. The substantial overlap among the descriptive profile groups does not support their interpretation as distinct biological phenotypes or as a diagnostic classification; the findings should therefore be considered exploratory and hypothesis-generating. Larger multicenter and longitudinal studies integrating standardized imaging, molecular biomarkers, and tissue assessment are needed to determine whether reproducible and clinically meaningful clinicopathological associations can be identified.

## Figures and Tables

**Table 1:** Radiographic characteristics.

Variable	Overall Cohort (*n* = 79)
Ahlbäck IV	28 (35.4%)
Ahlbäck V	51 (64.6%)
Absolute HKA deviation, degrees	6.2 ± 2.4
Varus alignment	51 (64.6%)
Valgus alignment	28 (35.4%)
Medial compartment predominance	48 (60.8%)
Lateral compartment predominance	25 (31.6%)
Patellofemoral compartment predominance	6 (7.6%)
Number of compartments involved	1.2 ± 0.4
Osteophyte burden	2.1 ± 0.9
Subchondral sclerosis	1.9 ± 1.0
Cartilage loss	2.5 ± 0.6
Compartment-specific overload	1.7 ± 0.9
Meniscal deficiency	1.6 ± 0.9
Focal cartilage defect	1.7 ± 0.9

HKA, hip–knee–ankle.

**Table 2:** Laboratory characteristics.

Variable	Overall Cohort (*n* = 79)
Inflammatory biomarkers	
C-reactive protein, mg/L	4.9 [2.6–7.5]
Erythrocyte sedimentation rate, mm/h	16.7 ± 7.7
Fibrinogen, g/L	3.24 ± 0.47
Neutrophil-to-lymphocyte ratio	2.07 [1.65–2.46]
Metabolic biomarkers	
HbA1c, %	5.4 [5.2–5.7]
Body mass index, kg/m^2^	30.0 ± 2.9
LDL cholesterol, mg/dL	105.7 ± 20.2
HDL cholesterol, mg/dL	52.2 ± 6.6
Triglycerides, mg/dL	136 [104–167]

**Table 3:** Unadjusted and age- and sex-adjusted associations between prespecified domain scores and synovial tissue outcomes.

Domain Score	Synovial Outcome	Spearman ρ	FDR-Adjusted q	Adjusted (95% CI)	Adjusted *p*	FDR-Adjusted q
Inflammatory	Krenn synovitis score	0.17	0.256	β = 0.24 (0.01 to 0.46)	0.043	0.100
Inflammatory	Macroscopic synovitis score	0.24	0.126	β = 0.23 (0.00 to 0.45)	0.050	0.100
Metabolic/lipomatous	Synovial lipomatosis severity	0.23	0.126	OR = 1.57 (1.01 to 2.42)	0.044	0.100
Osteocartilaginous/remodeling	Synovial remodeling score	0.01	0.916	β = −0.06 (−0.29 to 0.17)	0.605	0.605
Mechanical	Krenn synovitis score	−0.09	0.543	β = −0.10 (−0.34 to 0.14)	0.412	0.495
Mechanical	Macroscopic synovitis score	0.11	0.470	β = 0.18 (−0.05 to 0.42)	0.122	0.183

## Data Availability

The data presented in this study are available on request from the corresponding author due to privacy restrictions.

## Supplementary Materials

The following supporting information can be downloaded at https://www.mdpi.com/article/10.3390/diagnostics16213593/s1, Table S1: Definitions, coding, and data sources for the clinical and structural domain components and synovial outcomes; Figure S1: Mean standardized component values across the four descriptively classified dominant profile groups; Figure S2: Illustrative synovial histopathological features observed in patients from the four dominant clinical and structural profile groups; Table S2: Postoperative VAS and LEFS outcomes and exploratory associations of baseline clinical/structural domains and synovial tissue measures with 6-month postoperative outcomes; Table S3: Intraobserver agreement and reliability of histopathological and macroscopic synovial scoring; Table S4: Demographic, comorbidity, and medication characteristics across descriptive clinical and structural profile groups; Table S5: Sensitivity analyses of the primary continuous domain–tissue associations after adjustment for additional clinically relevant covariates.

## Abbreviations

The following abbreviations are used in this manuscript:

BMI	Body Mass Index
CI	Confidence Interval
CRP	C-Reactive Protein
ESR	Erythrocyte Sedimentation Rate
FDR	False Discovery Rate
HbA1c	Glycated Hemoglobin
HDL	High-Density Lipoprotein
HE	Hematoxylin and Eosin
HKA	Hip–Knee–Ankle
LDL	Low-Density Lipoprotein
MRI	Magnetic Resonance Imaging
OA	Osteoarthritis
OR	Odds Ratio
TKA	Total Knee Arthroplasty
VAS	Visual Analogue Scale
LEFS	Lower Extremity Functional Scale
NSAIDs	Nonsteroidal Anti-Inflammatory Drugs
