# Supplementary material for: Exploratory Clinicopathological Associations Between Prespecified Clinical–Structural Domains and Synovial Tissue Findings in End-Stage Knee Osteoarthritis
Source: Diagnostics (Basel). 2026 Oct 30;16(21):3593. doi: 10.3390/diagnostics16213593 (PMC13761913; doi:10.3390/diagnostics16213593)
Supplement: Supplementary file 1 [file diagnostics-16-03593-s001.zip › diagnostics-4505442-supplementary.pdf]

Table S1. Definitions, coding, and data sources for the clinical and structural domain components and synovial outcomes.

Domain/ outcome	Variable	Source	Coding/ unit
**Inflammatory domain**	C-reactive protein (CRP)	Preoperative laboratory test	mg/L; continuous
	Erythrocyte sedimentation rate (ESR)	Preoperative laboratory test	mm/h; continuous
	Fibrinogen	Preoperative laboratory test	g/L; continuous
	Neutrophil-to-lymphocyte ratio (NLR)	Preoperative complete blood count	Ratio; continuous
**Metabolic/lipomatous domain**	Body mass index (BMI)	Preoperative clinical assessment	kg/m²; continuous
	Glycated hemoglobin (HbA1c)	Preoperative laboratory test	%; continuous
	Triglycerides	Preoperative laboratory test	mg/dL; continuous
	Low-density lipoprotein cholesterol	Preoperative laboratory test	mg/dL; continuous
	High-density lipoprotein cholesterol	Preoperative laboratory test	mg/dL; reverse
**Osteocartilaginous/remodeling domain**	Ahlbäck grade	Preoperative radiograph	Ordinal grade; cohort restricted to grades IV-5
	Osteophyte burden	Preoperative imaging and operative documentation	Semiquantitative grade, 0-3
	Subchondral sclerosis	Preoperative imaging and operative documentation	Semiquantitative grade, 0-3
	Cartilage loss	Preoperative imaging and operative documentation	Semiquantitative grade, 0-3
	Number of compartments involved	Preoperative imaging and operative documentation	Numerical count
**Mechanical domain**	Absolute hip-knee-ankle deviation	Preoperative full-length alignment imaging	Degrees; continuous
	Compartment-specific overload	Preoperative imaging and operative documentation	Semiquantitative grade, 0-3
	Meniscal deficiency	Preoperative imaging and operative documentation	Semiquantitative grade, 0-3
	Focal cartilage defect	Preoperative imaging and operative documentation	Semiquantitative grade, 0-3
**Histopathological outcome**	Krenn lining hyperplasia	Suprapatellar synovial histology	0-3
	Krenn stromal cellularity	Suprapatellar synovial histology	0-3
	Krenn inflammatory infiltrate	Suprapatellar synovial histology	0-3
	Krenn total score	Sum of the three Krenn components	0-9; 0-1 absent, 2-4 low-grade, 5-9 high-grade
	Villous hyperplasia	Suprapatellar synovial histology	Absent (0), mild (1), moderate (2), marked (3)
	Vascular proliferation/congestion	Suprapatellar synovial histology	Absent (0), mild (1), moderate (2), marked (3)
	Fibrin/fibrinoid change	Suprapatellar synovial histology	Absent (0), mild (1), moderate (2), marked (3)
	Fibrosis	Suprapatellar synovial histology	Absent (0), mild (1), moderate (2), marked (3)
	Lipomatosis	Suprapatellar synovial histology	Absent (0), mild (1), moderate (2), marked (3)

	Calcification	Suprapatellar synovial histology	Absent (0), mild (1), moderate (2), marked (3)
	Chondrometaplasia	Suprapatellar synovial histology	Absent (0), mild (1), moderate (2), marked (3)
	Chondromatosis-like change	Suprapatellar synovial histology	Absent (0), mild (1), moderate (2), marked (3)
	Hemosiderin-rich macrophages/siderophages	Suprapatellar synovial histology	Absent (0), mild (1), moderate (2), marked (3)
	Reactive changes	Suprapatellar synovial histology	Absent (0), mild (1), moderate (2), marked (3)
	Synovial remodeling score	Sum of fibrosis, calcification, and chondrometaplasia scores	0-9
**Macroscopic outcome**	Synovial hypertrophy	Intraoperative assessment	Absent (0), mild (1), moderate (2), marked (3)
	Vascularity/hyperemia	Intraoperative assessment	Absent (0), mild (1), moderate (2), marked (3)
	Villous appearance	Intraoperative assessment	Absent (0), mild (1), moderate (2), marked (3)
	Pigmentation	Intraoperative assessment	Absent (0), mild (1), moderate (2), marked (3)
	Nodularity	Intraoperative assessment	Absent (0), mild (1), moderate (2), marked (3)
	Global macroscopic synovitis	Intraoperative assessment	Absent (0), mild (1), moderate (2), marked (3)
	Macroscopic synovitis total score	Sum of hypertrophy, vascularity, and villous-appearance scores	0-9

BMI, body mass index; CRP, C-reactive protein; ESR, erythrocyte sedimentation rate; HbA1c, glycated hemoglobin; HDL, high-density lipoprotein; HKA, hip-knee-ankle; LDL, low-density lipoprotein; NLR, neutrophil-to-lymphocyte ratio.

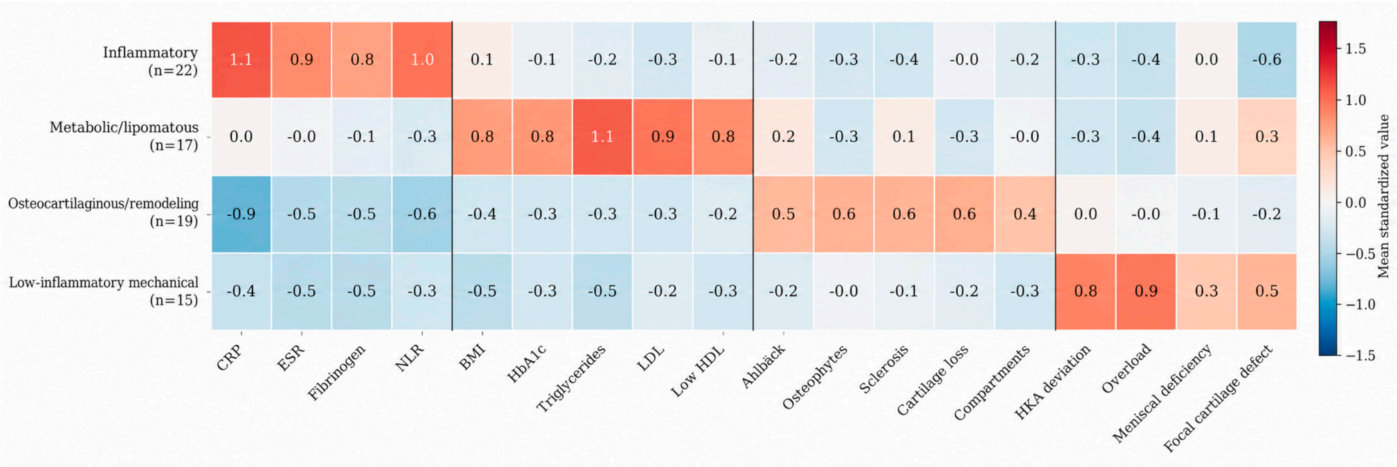

Figure S1. Mean standardized component values across the four descriptively classified dominant profile groups.

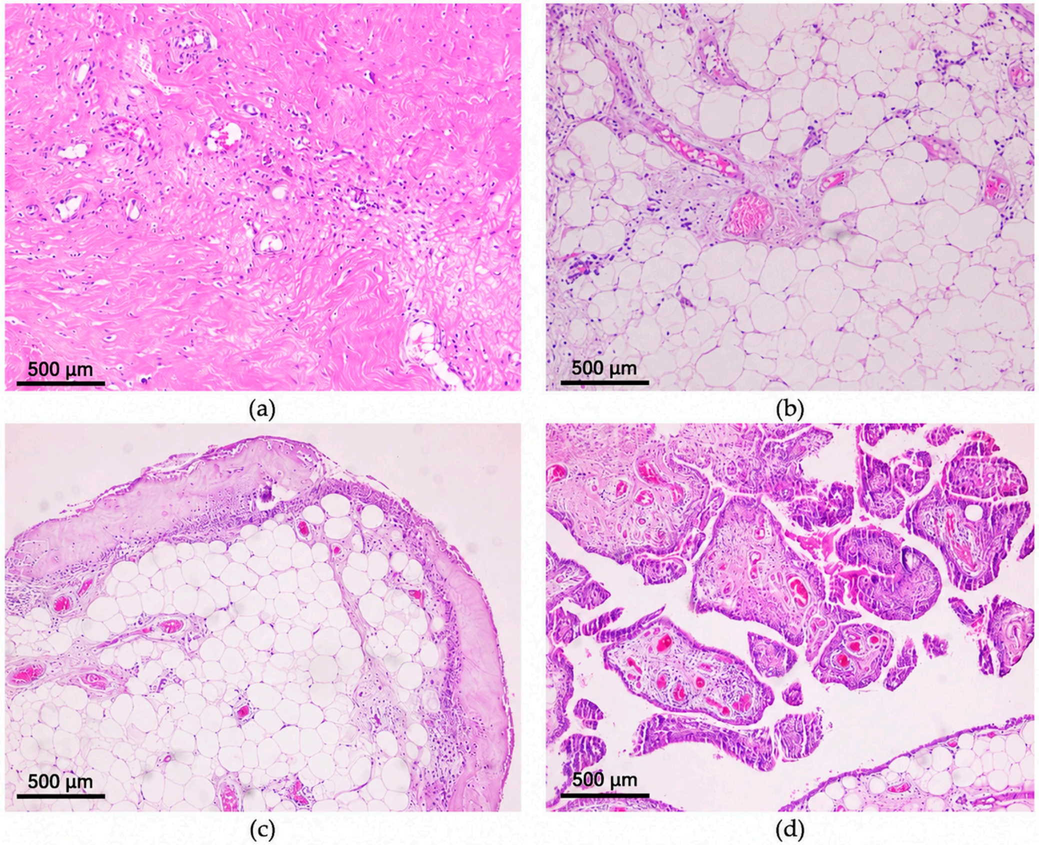

Figure S2. Illustrative synovial histopathological features observed in patients from the four dominant clinical and structural profile groups. The cases were selected from the four dominant descriptive profile groups; mixed profiles were not represented and histopathological findings were not used for profile assignment. Representative hematoxylin and eosin (HE)-stained sections from patients with end-stage knee OA. (a) Inflammatory: chronic synovitis with congested capillaries and dense chronic lymphoplasmacytic inflammatory infiltrate. (b) Metabolic/lipomatous: villous chronic synovitis with prominent lipomatous expansion of the villous core. (c) Osteocartilaginous/remodeling: villous chronic synovitis with cartilaginous metaplasia involving the synovial surface. (d) Low-inflammatory mechanical: preserved fibrovascular papillary architecture and micropapillary projections with only minimal inflammatory changes. Original magnification (5×). The images are for illustrative purposes only and should not be interpreted as evidence of biologically distinct or validated subtypes.

Table S2. Postoperative VAS and LEFS outcomes and exploratory associations of baseline clinical/structural domains and synovial tissue measures with 6-month postoperative outcomes

A. Postoperative VAS pain and LEFS scores at preoperative, 1-month, 3-month, and 6-month assessments.

Outcome	Preoperative	1 month (n=79)	3 months (n=79)	6 months (n=79)	Mean change, preop to 6 months
VAS pain score	6.80 ± 1.02	4.26 ± 1.18	2.48 ± 1.25	1.74 ± 0.96	5.06 ± 1.13
LEFS score	30.56 ± 8.16	37.75 ± 8.07	52.01 ± 8.78	61.42 ± 7.54	30.86 ± 8.54

For VAS, change was calculated as the preoperative score minus the 6-month score; for LEFS, change was calculated as the 6-month score minus the preoperative score. Positive values therefore indicate improvement

B. Exploratory baseline-adjusted associations with 6-month outcomes

Baseline measure	VAS at 6 months, standardized β (95% CI)	p	FDR q	LEFS at 6 months, standardized β (95% CI)	p	FDR q
Inflammatory domain	0.108 (−0.117 to 0.333)	0.341	0.867	−0.027 (−0.247 to 0.193)	0.806	0.962
Metabolic/lipomatous domain	0.004 (−0.230 to 0.237)	0.974	0.977	−0.071 (−0.287 to 0.145)	0.515	0.962
Osteocartilaginous/remodeling domain	−0.039 (−0.267 to 0.189)	0.736	0.962	−0.003 (−0.226 to 0.219)	0.977	0.977
Mechanical domain	−0.234 (−0.455 to −0.012)	0.039	0.626	0.166 (−0.051 to 0.382)	0.131	0.867
Krenn synovitis score	0.122 (−0.094 to 0.339)	0.265	0.867	−0.044 (−0.255 to 0.167)	0.676	0.962
Macroscopic synovitis score	0.118 (−0.099 to 0.336)	0.282	0.867	−0.140 (−0.351 to 0.070)	0.188	0.867
Synovial lipomatosis severity	−0.097 (−0.316 to 0.122)	0.379	0.867	0.057 (−0.154 to 0.268)	0.592	0.962
Synovial remodeling score	0.023 (−0.206 to 0.253)	0.841	0.962	−0.056 (−0.278 to 0.165)	0.613	0.962

Panel B models include the corresponding preoperative outcome, age, and sex as covariates. Benjamini-Hochberg FDR correction was applied across the 16 exploratory postoperative association tests

Table S3. Intraobserver agreement and reliability of histopathological and macroscopic synovial scoring

Assessment	Agreement (%)	Reliability statistic (95% CI)
**Histopathological assessment**		
Synovial lining hyperplasia	90.0	κ = 0.86 (0.73-0.95)
Stromal cellularity	86.7	κ = 0.81 (0.66-0.92)

Inflammatory infiltrate	90.0	κ = 0.85 (0.72-0.94)
Krenn synovitis score	—	ICC = 0.92 (0.84-0.96)
Villous hyperplasia	83.3	κ = 0.78 (0.61-0.89)
Vascular proliferation/congestion	86.7	κ = 0.82 (0.67-0.92)
Fibrosis	90.0	κ = 0.87 (0.74-0.95)
Lipomatosis	93.3	κ = 0.89 (0.76-0.96)
Calcification	93.3	κ = 0.76 (0.55-0.89)
Chondrometaplasia	86.7	κ = 0.79 (0.62-0.90)
Chondromatosis-like change	93.3	κ = 0.74 (0.52-0.88)
Fibrin/fibrinoid deposition	86.7	κ = 0.80 (0.64-0.91)
Hemosiderin-rich macrophages	90.0	κ = 0.83 (0.68-0.93)
Reactive synovial changes	83.3	κ = 0.77 (0.59-0.89)
**Synovial remodeling score**	—	ICC = 0.90 (0.80-0.95)
**Macroscopic assessment**		
Synovial hypertrophy	86.7	κ = 0.82 (0.67-0.92)
Vascularity	83.3	κ = 0.78 (0.60-0.89)
Villous appearance	80.0	κ = 0.73 (0.54-0.86)
Pigmentation	90.0	κ = 0.85 (0.70-0.94)
Nodularity	83.3	κ = 0.76 (0.58-0.88)
Global macroscopic synovitis	86.7	κ = 0.81 (0.65-0.91)
**Macroscopic synovitis score**	—	ICC = 0.89 (0.79-0.95)

Table S4. Demographic, comorbidity and medication characteristics across descriptive clinical and structural profile groups

**Characteristic**	**Overall (n=79)**	**Inflammatory (n=22)**	**Metabolic/lipomatous (n=17)**	**Osteocartilaginous/remodeling (n=19)**	**Low-inflammatory mechanical (n=15)**	**Mixed (n=6)**	**p value**

Age, years	61.5 ± 8.8	61.1 ± 7.9	57.5 ± 9.3	64.4 ± 7.7	63.6 ± 10.3	59.3 ± 6.2	0.076
Female sex	45 (57.0%)	11 (50.0%)	11 (64.7%)	14 (73.7%)	8 (53.3%)	1 (16.7%)	0.437
Hypertension	44 (55.7%)	9 (40.9%)	12 (70.6%)	9 (47.4%)	9 (60.0%)	5 (83.3%)	0.279
Diabetes mellitus	10 (12.7%)	4 (18.2%)	1 (5.9%)	3 (15.8%)	1 (6.7%)	1 (16.7%)	0.615
Dyslipidemia	22 (27.8%)	8 (36.4%)	7 (41.2%)	3 (15.8%)	2 (13.3%)	2 (33.3%)	0.16
Current smoking	12 (15.2%)	5 (22.7%)	1 (5.9%)	3 (15.8%)	3 (20.0%)	0 (0.0%)	0.546
NSAID use	34 (43.0%)	8 (36.4%)	9 (52.9%)	10 (52.6%)	6 (40.0%)	1 (16.7%)	0.634
Statin use	30 (38.0%)	10 (45.5%)	6 (35.3%)	8 (42.1%)	4 (26.7%)	2 (33.3%)	0.689
Antidiabetic medication	7 (8.9%)	3 (13.6%)	0 (0.0%)	2 (10.5%)	1 (6.7%)	1 (16.7%)	0.512
Antiplatelet use	20 (25.3%)	5 (22.7%)	4 (23.5%)	4 (21.1%)	6 (40.0%)	1 (16.7%)	0.625
Anticoagulant use	10 (12.7%)	2 (9.1%)	1 (5.9%)	2 (10.5%)	3 (20.0%)	2 (33.3%)	0.682

Table S5. Sensitivity analyses of the primary continuous domain-tissue associations after adjustment for additional clinically relevant covariates.

Association	Age- and sex-adjusted estimate (95% CI)	Additional covariates	Sensitivity-adjusted estimate (95% CI)	p	FDR q
Inflammatory domain - Krenn synovitis	β = 0.236 (0.008 to 0.463)	BMI, current smoking, NSAID use	β = 0.207 (−0.034 to 0.448)	0.091	0.182
Inflammatory domain - Macroscopic synovitis	β = 0.227 (0.000 to 0.454)	BMI, current smoking, NSAID use	β = 0.228 (−0.010 to 0.466)	0.060	0.180

Metabolic/lipomatous domain - Synovial lipomatosis	OR = 1.56 (1.01 to 2.41)	Hypertension, current smoking, statin use	OR = 1.89 (1.18 to 3.05)	0.009	0.052
Osteocartilaginous/remodeling domain - Synovial remodeling	β = −0.060 (−0.289 to 0.170)	BMI, current smoking, diabetes mellitus	β = −0.087 (−0.314 to 0.141)	0.450	0.469
Mechanical domain - Krenn synovitis	β = −0.098 (−0.337 to 0.140)	BMI, current smoking, NSAID use	β = −0.089 (−0.331 to 0.154)	0.469	0.469
Mechanical domain - Macroscopic synovitis	β = 0.184 (−0.050 to 0.418)	BMI, current smoking, NSAID use	β = 0.184 (−0.053 to 0.422)	0.126	0.190

Sensitivity models retained age and sex from the primary adjusted analyses. Additional covariates were BMI, current smoking, and NSAID use for the inflammatory and mechanical domain models; hypertension, current smoking, and statin use for the metabolic/lipomatous domain model; and BMI, current smoking, and diabetes mellitus for the osteocartilaginous/remodeling domain model. Variables constituting a given domain score were not additionally entered as covariates in that domain’s sensitivity model. Benjamini-Hochberg FDR correction was applied across the six sensitivity analyses.
